# Near-isogenic lines of *Triticum aestivum* with distinct modes of resistance exhibit dissimilar transcriptional regulation during *Diuraphis noxia* feeding

**DOI:** 10.1242/bio.201410280

**Published:** 2014-10-31

**Authors:** Anna-Maria Botha, Leon van Eck, N. Francois V. Burger, Zacharias H. Swanevelder

**Affiliations:** 1 Department of Genetics, Stellenbosch University, Stellenbosch 7601, South Africa; 2 ARC-Biotechnology Platform, Agricultural Research Council, Onderstepoort 0110, South Africa; 3 University of Minnesota, Saint Paul, Minneapolis, MN 55455-0213, USA

**Keywords:** Aphid feeding, cDNA-AFLP, Expression profiling, Peroxidase, Glutathione-*S*-transferase, Lipoxygenase, *β*-1,3-glucanase, Affymetrix

## Abstract

Russian wheat aphid (*Diuraphis noxia*, Kurdjumov) feeding on susceptible *Triticum aestivum* L. leads to leaf rolling, chlorosis and plant death – symptoms not present in resistant lines. Although the effects of several *D. noxia* (*Dn*) resistance genes are known, none have been isolated or characterized. Wheat varieties expressing different *Dn* genes exhibit distinct modes of *D. noxia* resistance, such as antibiosis (*Dn1*), tolerance (*Dn2*), and antixenosis (*Dn5*). However, the mechanism whereby feeding aphids are perceived, and how subsequent transcriptional responses are partitioned into resistance categories, remains unclear. Here we report on downstream events in near-isogenic wheat lines containing different *Dn* genes after *D. noxia* biotype SA1 feeding. Transcripts involved in stress, signal transduction, photosynthesis, metabolism and gene regulation were differentially regulated during *D. noxia* feeding. Expression analyses using RT-qPCR and RNA hybridization, as well as enzyme activity profiling, provide evidence that the timing and intensity of pathways induced are critical in the development of particular modes of resistance. Pathways involved include the generation of kinase signalling cascades that lead to a sustained oxidative burst, and a hypersensitive response that is active during antibiosis. Tolerance is a passive resistance mechanism that acts through repair or *de novo* synthesis of photosystem proteins. Results further suggest that ethylene-mediated pathways are possibly involved in generating volatile compounds and cell wall fortification during the antixenosic response.

## INTRODUCTION

Aphids are the largest group of phloem-feeding insects and their enormous reproductive potential makes them some of the most devastating pests to crop production (Davis, 2012). Aphids have evolved a more intimate association with their plant hosts than herbivorous insects, eliciting the expression of plant genes commonly associated with bacterial and fungal pathogen attack (Moran and Thompson, 2001; Botha et al., 2010; Smith et al., 2010). The interaction between wheat (*Triticum aestivum* L.) and *Diuraphis noxia* (Kurdjumov), commonly known as the Russian wheat aphid, has been of major interest to researchers in this field, particularly the identity and function of *D. noxia* effectors and wheat resistance genes.

**Fig. 1.**
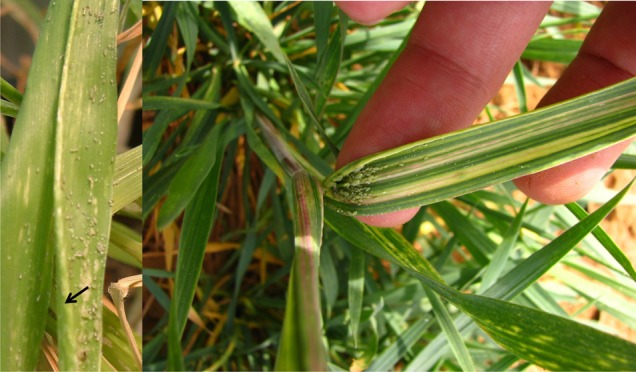
Symptoms of *D. noxia* infestation on susceptible wheat, indicating leaf rolling (arrow) (*left*), purplish streaking and chlorosis (*right*).

During compatible interactions with susceptible wheat cultivars, *Diuraphis noxia* feeding interferes with the osmoregulation of leaf turgor pressure during cell elongation (Burd and Burton, 1992), preventing the proper unfolding of new leaves (Fig. 1). Additionally, feeding causes chlorosis and longitudinal streaking, reducing leaf chlorophyll content (Fig. 1) (Heng-Moss et al., 2003; Botha et al., 2005; Botha et al., 2006). The result is decreased photosynthetic potential and the eventual collapse of the plant (Burd and Burton, 1992). This has historically been ascribed to a phytotoxin injected during feeding, presumed responsible for chloroplast disintegration (Fouché et al., 1984), but such a phytotoxic effector has never been isolated or characterized. Current hypotheses suggest that *D. noxia* feeding induces malfunctioning of the photosynthetic apparatus at the stacked region of the thylakoid membrane, although the exact site of interference has not been determined either (Burd and Elliott, 1996; Heng-Moss et al., 2003). However, *D. noxia* feeding does not induce total breakdown of the chloroplast, (Haile et al., 1999; van der Westhuizen et al., 1998a) and feeding-induced chlorosis differs from normal chlorophyll degradation that occurs during leaf senescence (Ni et al., 2001; Wang et al., 2004a).

An arsenal of eleven wheat *Dn* genes (*Dn1-Dn9*, *Dnx* and *Dny*) has been described that confer resistance against *D. noxia*. These are hypothesized to function much like classic pathogen resistance genes, by encoding proteins that recognize aphid-specific effectors in a gene-for-gene manner and then initiate signalling cascades resulting in a defence response (Flor, 1971; Lacock et al., 2003; Botha et al., 2005; Lapitan et al., 2007). However, none of the *Dn* genes have been cloned, and results from mapping efforts are often contradictory or inconclusive (Ma et al., 1998; Myburg et al., 1998; Liu et al., 2001; Heyns et al., 2006). *Dn1*, *Dn2* and *Dn5* are located on chromosome 7D, but whether they are allelic at the same locus or independent, but tightly linked, is unclear (Liu et al., 2001; Heyns et al., 2006). Some heterogeneity in the original PI 294994 accession from which *Dn5* was acquired, may explain why conflicting results were obtained by different research groups (Marais and Du Toit, 1993; Zhang et al., 1998). The genetic background in which a specific *Dn* gene is bred may also play a role in the successful establishment of a resistant phenotype, impeding their characterization (van der Westhuizen et al., 1998a; van der Westhuizen et al., 1998b). For example, although the presence of the *Dn1* gene does not make the cultivar Betta-*Dn1* less inclined to aphid-induced chlorophyll loss compared to its susceptible near-isogenic line (NIL) Betta (Heng-Moss et al., 2003), the presence of *Dn1* in the cultivar Tugela-*Dn1* does prevent chlorophyll levels from decreasing to levels observed in its susceptible NIL, Tugela (Botha et al., 2006).

The resistance mediated by various *Dn* genes can be phenotypically categorized as antibiosis, antixenosis, and tolerance (Painter, 1951; Painter, 1958). A resistant cultivar may exhibit a combination of these categories of resistance (Haile et al., 1999; Smith et al., 1992), such as those bred to contain *Dn5*, which affords a combination of antibiosis and antixenosis (Wang et al., 2004b). Antibiosis is observed when the plant reduces the reproductive fitness of aphids feeding on it and several studies indicate that *Dn1* affords antibiosis (Du Toit, 1989; Smith et al., 1992; Unger and Quisenberry, 1997; Budak et al., 1999; Wang et al., 2004b). Tolerance is seen as a lack of plant height reduction despite feeding, and is the primary resistance category described for *Dn2* (Du Toit, 1989; Budak et al., 1999; Wang et al., 2004b). Antixenosis is the non-preference of a cultivar as host, and in addition to moderate antibiosis, the *Dn5* gene affords the antixenotic phenotype (Du Toit, 1989; Smith et al., 1992; Marais and Du Toit, 1993; Wang et al., 2004b).

Although the effects of these *Dn* genes on aphid reproduction have been well-characterized, aphid-induced transcriptional reprogramming within wheat lines expressing these resistance genes is still poorly understood. In order to shed light on the specific genetic pathways underlying each phenotypic category of resistance we investigated differential wheat gene expression associated with the generation of antibiotic, antixenotic, or tolerance resistance responses to *D. noxia* feeding in Tugela NILs containing different *Dn* genes.

## RESULTS

### cDNA-AFLP transcript profiling

Following the cDNA-AFLP analysis approach, we were able to excise forty-nine differentially regulated TDFs (supplementary material Table S1). After the sequences were obtained, putative identities were assigned to the clones using BLASTx or BLASTn. Based on the putative functions of the proteins inferred by similarity, the TDFs were classified into five broad functional categories (supplementary material Fig. S1). Of the total number of sequenced TDFs, 14% were involved in general gene regulation and metabolism, 25% in stress and signal transduction and 12% in photosynthesis. The remainder of TDFs either exhibited similarity to hypothetical proteins or proteins of unknown function, or were classified as TDFs with no significant similarity to proteins in the non-redundant database. Novel sequences were entered into the GenBank EST database. TDFs categorized as regulatory transcripts included a lingual lipase-like gene, TPA cysteine protease, a putative transfactor, a methyl CpG-binding protein, ethylene-responsive RNA helicase, C4-type zinc finger protein, ubiquitin and ubiquitin-protein ligase I (supplementary material Table S1). The stress and signal transduction category included such diverse transcripts as a mitochondrial half-ABC transporter, a mechano-sensitive ion channel protein, kinases (i.e. GHMP kinase and serine/threonine protein kinase), inorganic pyrophosphatase, a stress related-like protein interactor, isomerases (i.e. PDI-1 protein disulfide isomerase 1 and IDI2 isopentenyl-diphosphate delta isomerase 2), a 66 kDa stress protein and KCO1 outward-rectifying potassium channel. Several TDFs grouped together functionally as components of photosynthesis. This category clearly indicated the importance of the Rubisco small subunit during the wheat response to *D. noxia* (van der Westhuizen and Botha, 1993), with three of the TDFs obtained showing such similarity. Other transcripts included a TMP 14 kDa thylakoid membrane phosphoprotein, fructose-1,6-*bis*phosphatase and aconitate hydratase.

The results from select candidates obtained using the AFLP transcriptional profiling were verified using RT-qPCR and slot-blot RNA hybridization (supplementary material Fig. S2). In all three cases, band intensities indicated similar trends to those obtained with RT-qPCR.

### GeneChip Wheat Genome Array transcript profiling

From the Affymetrix arrays, most of the transcriptional changes observed during *D. noxia* infestation of wheat were involved in the suppression rather than the induction of genes, with a total of 5649 genes that were down-regulated, while 5468 genes were up-regulated (supplementary material Fig. S3A,B). Of these, a total of 4306 genes were shared by all the genotypes. The obtained genes were assigned to broad functional categories and their involvement in metabolism (supplementary material Tables S2, S3). Most of the genes that were differentially regulated irrespective of genotype, belonged to the undescribed or unknown categories.

Of the genes that could be assigned to a functional group or metabolism, most of the genes belong to the carbohydrate metabolism category, and are thus involved in carbon flux. Other observable differences include a higher number of genes involved in energy metabolism. Genes belonging to carbohydrate metabolism and cell wall synthesis that were well represented include several copies of probe sets with high similarity to glucan 1,3-beta-glucosidase, L-allo-threonine aldolase, Phosphoglycerate dehydrogenase, pyruvate kinase, phosphoglycerate dehydrogenase, cellulose synthase, phosphatephosphoenolpyruvate, hexokinase, trehalose-6-phosphate synthase, GDP-mannose pyrophosphorylase, sugar transporters, extracellular invertase.

Well-represented genes involved in photosynthesis, starch synthesis or that are chloroplast-related included probe sets with high similarity to photosystem II type I chlorophyll a b binding protein, thioredoxin f1, chlorophyll synthetase, fructose 1,6-bisphosphatase, chloroplast 50S ribosomal protein, ATP-dependent Clp protease proteolytic subunit, non-green plastid inner envelope, ferredoxin–thioredoxin reductase and photolyase blue-light receptor.

Defence and stress-related genes included several copies of probe sets with high similarity to viral resistance protein, Pto kinase interactor, thaumatin, phloem-specific lectin, 12-oxophytodienoate reductase, heat shock factor protein hsfβ, salt-tolerance protein, biostress-resistance-related protein, metalloprotease, selenium-binding protein, SAR DNA-binding protein-1 and controlled tumour protein-like protein.

Genes involved in lipid and fatty acid metabolism were also well represented and included several copies of lipid-transfer proteins, glyoxalase 1, glycerophosphodiester phosphodiesterase, (acyl-carrier-protein) S-malonyltransferase, lipophosphoglycan biosynthetic protein, phosphocholine cytidylyltransferase, 3-hydroxybutyryl-CoA dehydrogenase and protein phosphatase type 2C proteins.

Signal transduction-genes were well represented in the study and include several copies of serine/threonine-specific protein kinases, postsynaptic protein CRIPT, ras-related small GTP-binding protein RAB1c and Rab11, RAN2 small Ras-like GTP-binding nuclear protein, GTP-binding proteins, GTP cyclohydrolases, protein kinases, AMP-binding proteins, and ABC transporter-like proteins.

Genes involved in proton pumps and Ca^2+^ transport included several copies of potassium transporters, calmodulin 6 and Myo-inositol 1-phosphate synthase-like proteins, as well as endomembrane and integral membrane proteins, a phosphoinositide-specific phospholipase, phosphatidylinositol-4-phosphate 5-kinases, importin beta, and calcium-binding proteins.

### Genotype specific signatures

When comparing the expression of genes between genotypes, it was apparent that genotype specific differential expression patterns could be discerned (supplementary material Table S3). In the susceptible Tugela and the tolerant Tugela-*Dn2*, genes related to stress, proton pumps and Ca^2+^ transport, protein biosynthesis and modification, cell cycle regulation, and cellular respiration were differentially regulated. Genes significantly up-regulated in the susceptible Tugela but not in Tugela-*Dn2*, included senescence associated genes (i.e., a decay protein, an auxin-responsive protein, senescence-specific cysteine protease SAG12, senescence-associated proteins), carboxylesterases involved in ROS production, and a ras-related small GTP-binding protein RAB1c involved in cell signaling. While carbon flux and photosynthesis related genes (i.e., ferredoxin–thioredoxin reductase, fructose 1,6-bisphosphatase, chloroplast 50S ribosomal protein, ATP-dependent Clp protease proteolytic subunit) were up-regulated only in the tolerant Tugela-*Dn2*.

Similar general trends in the regulation of genes belonging to specific functional categories were observed in the antibiotic (Tugela-*Dn1*) and antixenotic (Tugela-*Dn5*) genotypes. Transcripts involved in the metabolism of cofactors, vitamins, secondary metabolites, transcription factors (e.g. MADS-box TFs and T48034 bZIP transcription factor-like proteins), defence, as well as ROS and signal transduction were differentially regulated in both genotypes. However, the photosystem II type I chlorophyll a b binding protein and thioredoxin f1 were only significantly up-regulated in the antibiotic Tugela-*Dn1*, while the non-green plastid inner envelope, chlorophyll synthetase and photolyase blue-light receptor were only up-regulated in the antixenotic Tugela-*Dn5*. Interestingly, genes belonging to secondary metabolism and ROS that were differentially regulated only in the antixenotic Tugela-*Dn5*, included glutathione S-transferase (significantly up-regulated) and manganese superoxide dismutase (SOD) (significantly down-regulated); cellulose synthase involved in cell wall synthesis and phosphocholine cytidylyltransferase in membrane synthesis (significantly up-regulated); monooxygenase, β-glucosidase and O-methyltransferases involved in the production of VOCs (significantly up-regulated); and a viral resistance protein and Pto kinase interactor (significantly up-regulated).

Genes that were differentially regulated in the antibiotic Tugela-*Dn1* and tolerant Tugela-*Dn2*, but not the antixenotic Tugela-*Dn5* included genes involved in ethylene biosynthesis (i.e., 1-aminocyclopropane-1-carboxylate oxidase), SAR DNA-binding protein-1 and a serine/threonine-specific protein kinases involved in cell signaling.

### Enzyme activities in response to *D. noxia* feeding

**Fig. 2.**
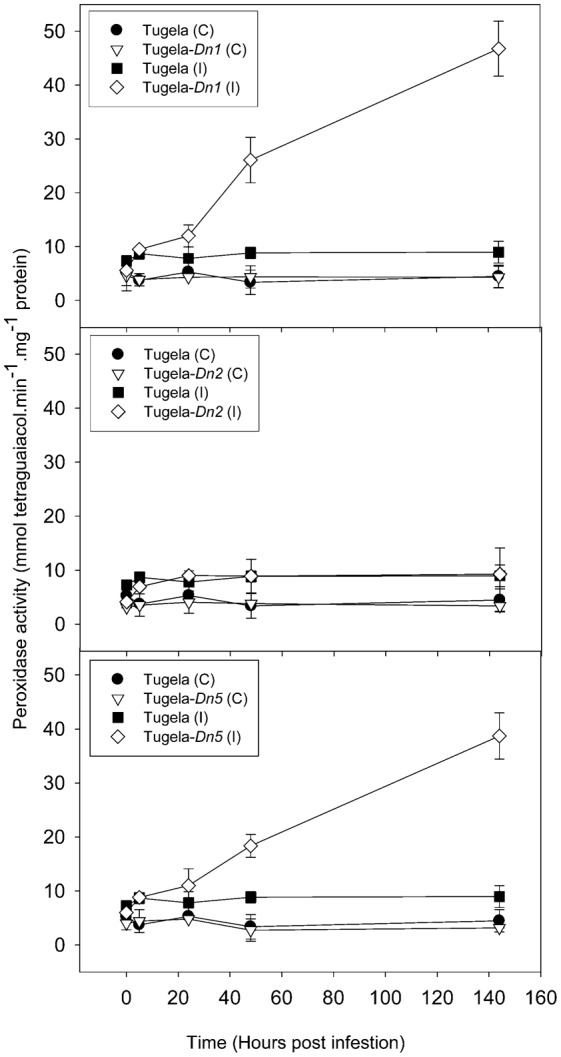
Effect of *D. noxia* infestation on total peroxidase activity of susceptible (Tugela) and resistant (Tugela-*Dn1*, Tugela-*Dn2*, Tugela-*Dn5*) near isogenic wheat lines. The formation of tetraguaiacol was monitored at 470 nm. Values are means ± SD (*n* = 3).

**Fig. 3.**
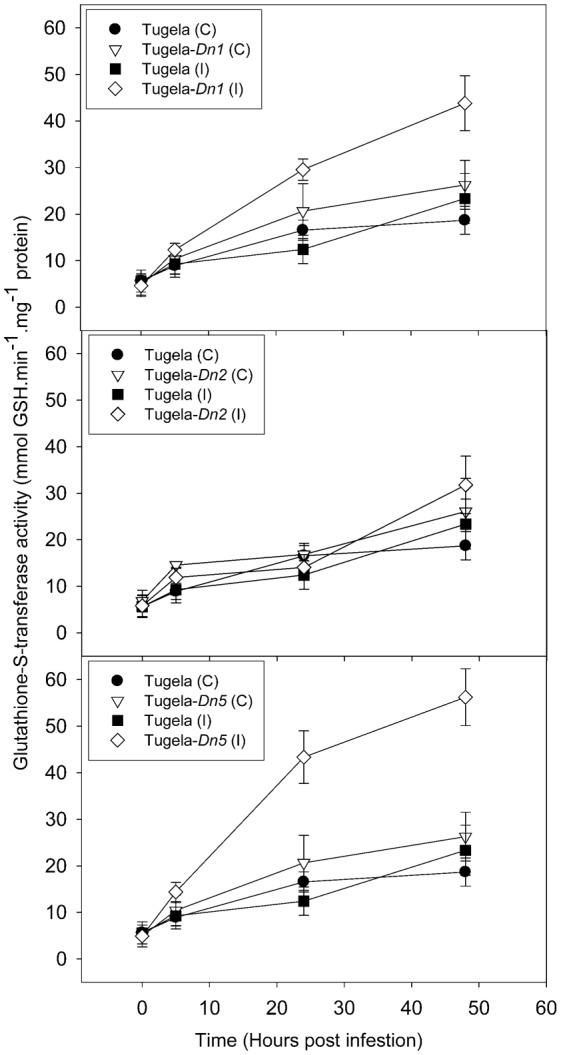
Effect of *D. noxia* infestation on total glutathione-S-transferase (GST) activity of susceptible (Tugela) and resistant (Tugela-*Dn1*, Tugela-*Dn2*, Tugela-*Dn5*) near isogenic wheat lines. The formation of GS-DNB conjugate was monitored at 340 nm. Values are means ± SD (*n* = 3).

**Fig. 4.**
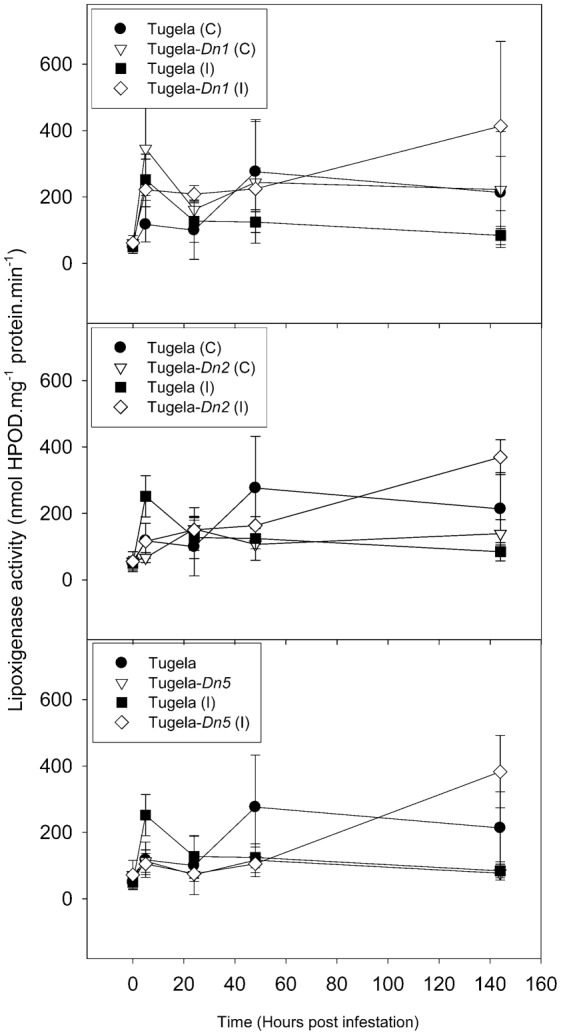
Effect of *D. noxia* infestation on total lipoxygenase (LOX) activity of susceptible (Tugela) and resistant (Tugela-*Dn1*, Tugela-*Dn2*, Tugela-*Dn5*) near isogenic wheat lines. Values are means ± SD (*n* = 3).

**Fig. 5.**
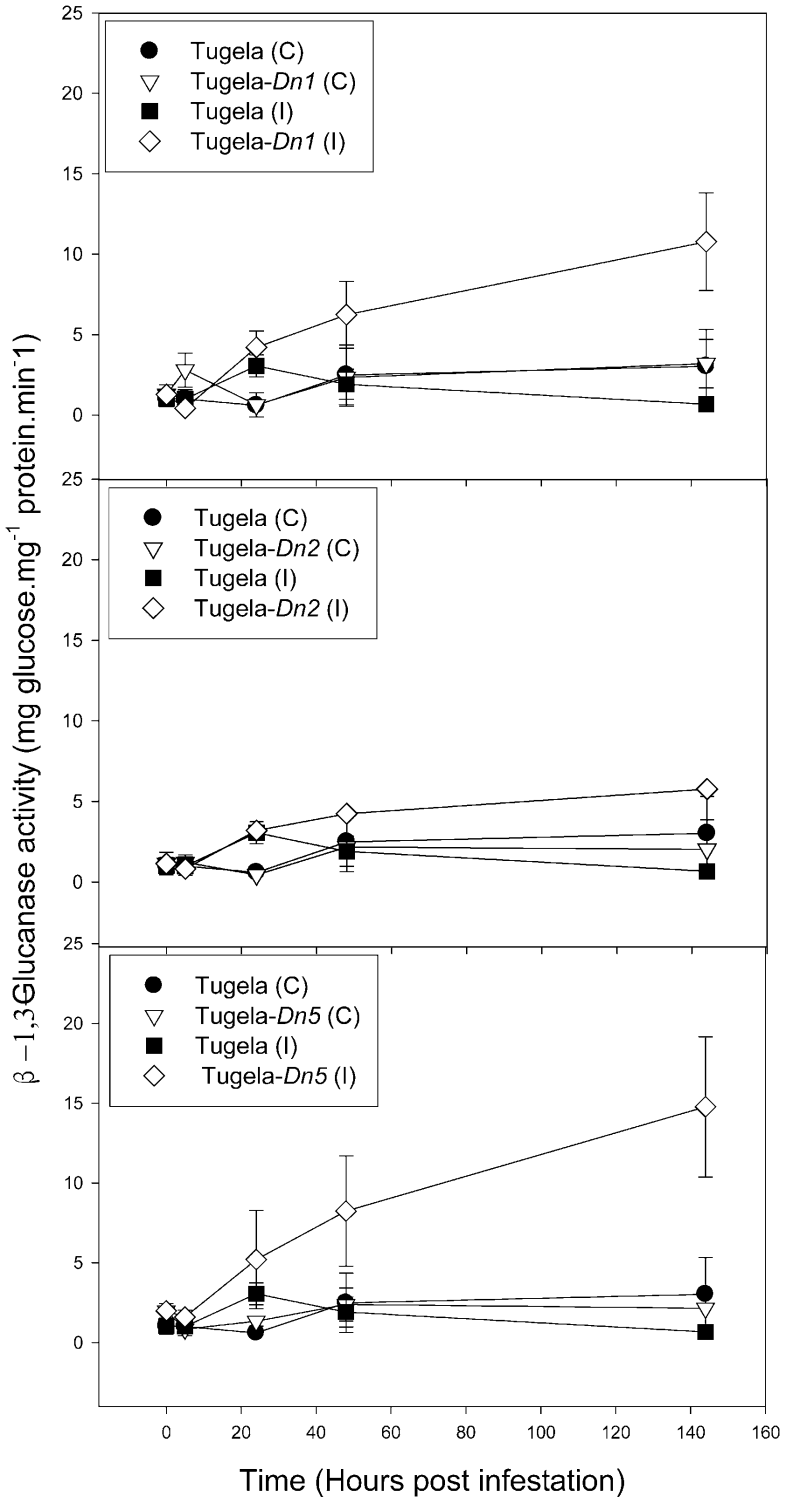
Effect of *D. noxia* infestation on the total β-1,3-glucanase activity of susceptible (Tugela) and resistant (Tugela-*Dn1*, Tugela-*Dn2*, Tugela-*Dn5*) near isogenic wheat lines. Values are means ± SD (*n* = 3).

To elucidate the effect of *D. noxia* feeding on the NILs, levels of ROS enzymes (peroxidase, LOX and GST) and a SAR marker enzyme (β-1,3-glucanase) were measured in uninfested leaf tissue (Figs 2–5). The activity of peroxidase and GST were significantly higher in the resistant NILsTugela-*Dn1* and Tugela-*Dn5* after infestation with *D. noxia* when compared with susceptible Tugela and the tolerant Tugela-*Dn2* (Figs 2 and 3). Even though LOX activity was higher in the resistant NILs when compared with the susceptible Tugela, the activity did not differ significantly (*P*≤0.05) (Fig. 4). Glucanase activity increased in the resistant NILs when compared to the susceptible Tugela after *D. noxia* feeding (Fig. 5).

### Oxidative burst in response to *D. noxia* feeding

**Fig. 6.**
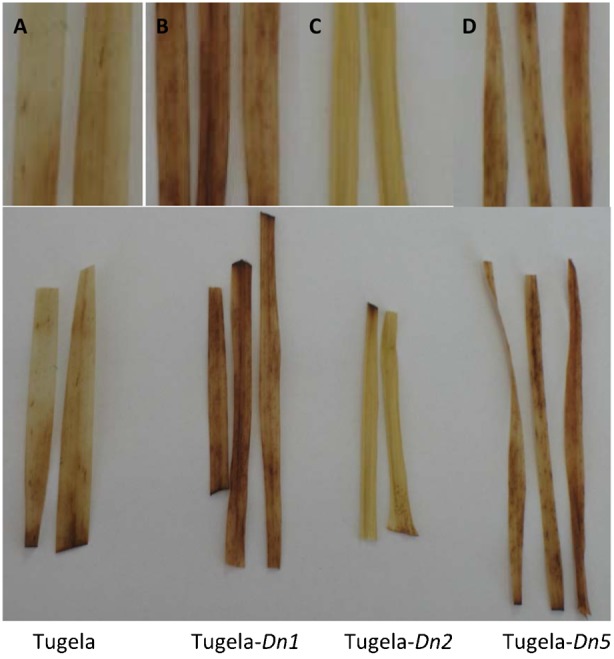
*D. noxia* feeding results in peroxide accumulation at aphid feeding sites after staining leaves of infested near isogenic wheat lines plants with 3,3′-diaminobenzidine (DAB) 132 hpi. Leaves are representative of five independent biological replicates per treatment. (A) Tugela; (B) Tugela-*Dn1* (antibiotic); (C) Tugela-*Dn2* (tolerant); (D) Tugela-*Dn5* (antixenotic). Tugela-*Dn1* (B) and Tugela-*Dn5* (D) show dark areas around aphid feeding sites indicating the presence of peroxide. Tugela (A) and Tugela-*Dn2* (C) only show dark areas at cut ends of the leaves.

To corroborate the changes in the oxidative burst in the NILs in response to *D. noxia* biotype SA1 feeding, leaves were collected and stained for H_2_O_2_ using 3,3′-diaminobenzidine (DAB), which forms reddish-brown polymerized deposits in the presence of peroxidase (Fig. 6). Dark staining was observed in Tugela-*Dn1* and Tugela-*Dn5* around the feeding sites (Fig. 6B and Fig. 5D), but staining was only visible around the regions where the leaves were cut in Tugela and Tugela-*Dn2* (Fig. 6A,C). This indicates that the generation of aphid-induced ROS was active only in the NILs associated with antibiotic and antixenotic resistance responses, but not the susceptible Tugela nor the tolerant Tugela-*Dn2*.

## DISCUSSION

Effective wheat host defence responses against *D. noxia* occur via different modes of resistance: antibiosis, antixenosis, tolerance, or a combination of these (Painter, 1951; Painter, 1958). These must be mediated by specific genetic pathways, and signal transduction, ethylene-mediated responses and systemic resistance have been described as possible ways to counteract the attack by *D. noxia* (Botha et al., 1998; Botha et al., 2005; Botha et al., 2006; Botha et al., 2010; Boyko et al., 2006; Smith and Boyko, 2007; Smith et al., 2005; Smith et al., 2010; Van Eck et al., 2010; Marimuthu and Smith, 2012; Liu et al., 2011). In order for the host to sustain growth and proliferate under attack by phloem feeding insects, it must be able to recognize the invasion and initiate a defensive response. For this, the host must utilize an effective signaling cascade to initiate defensive syndromes to ensure survival (van der Westhuizen et al., 1998a; van der Westhuizen et al., 1998b; Botha et al., 1998; Botha et al., 2005; Botha et al., 2006; Botha et al., 2010; Boyko et al., 2006; Smith et al., 2005; Smith et al., 2010; Smith and Boyko, 2007; Van Eck et al., 2010; Marimuthu and Smith, 2012). Failure to do so is associated with elevated stress and early onset of senescence and under severe infestations, even death, as observed in the susceptible Tugela cultivar. *Diuraphis noxia* feeding elicits salicylic acid (SA) and jasmonic acid/ethylene (JA/Eth) signalling pathways during mobilization of defensive strategies against aphids as shown by the induction of transcripts associated with these plant hormones (Miller et al., 1994; Botha et al., 1998; Moran and Thompson, 2001; Voelckel et al., 2004; De Vos et al., 2005; Divol et al., 2005; Mewis et al., 2005; Zhu-Salzman et al., 2005; Thompson and Goggin, 2006; Couldridge et al., 2007; Kuśnierczyk et al., 2008; Gutsche et al., 2009; Botha et al., 2010). Measurement of β-1,3-glucanase activity (a SAR associated enzyme) showed significant differences in the activity of this enzyme between the resistant NILs and the susceptible Tugela (van der Westhuizen et al., 1998b), which ensured sustained long-term systemic acquired resistance.

### Antibiosis is likely due to an oxidative burst and subsequent hypersensitive response

In resistant varieties, the early (within the first 5 hpi) up-regulation of transcripts associated with an increase in cytosolic Ca^2+^ (Czempinski et al., 1997; Bouché et al., 2005; Ma and Berkowitz, 2007) have been shown to accompany *D. noxia* feeding (Botha et al., 2010). This process may initiate long-distance calcium-activated protein kinase signalling cascades (Kehr, 2006) transmitting primary recognition responses to multiple downstream effectors, including activation of the oxidative burst (elevated H_2_O_2_) (Foyer and Noctor, 2005; Foyer and Noctor, 2009) to induce cell death or necrosis (van der Westhuizen et al., 1998a; van der Westhuizen et al., 1998b; Boyko et al., 2006; Botha et al., 2005; Botha et al., 2006; Botha et al., 2010; Smith et al., 2005; Smith and Boyko, 2007) and the hypersensitive response (HR) (Grant et al., 2000; Botha et al., 2005; Botha et al., 2010; Smith et al., 2005; Smith et al., 2010) during incompatible interactions. Signalling cascades are well described in the plant pathology literature (Hammond-Kosack and Jones, 1996; Dangl and Jones, 2001; Lam et al., 2001), and these are especially prominent in the antibiotic and antixenotic cultivars. Signalling cascades activate downstream proteins through phosphorylation, propagating a recognition signal that eventually leads to defence responses, most often in the form of a HR. Indeed, *D. noxia* feeding on antibiotic cultivars, like those containing *Dn1*, initiates an HR closely resembling that observed during plant–pathogen interactions. HR is associated with the production of reactive oxygen species (ROS), like hydrogen peroxide (H_2_O_2_), and programmed cell death at the site of aphid probing. This is analogous to events at the site of hyphal penetration by pathogenic fungi or during bacterial ingress. ROS also induce the accumulation of salicylic acid, which in turn stimulates the expression of pathogenesis-related (PR) proteins like chitinases (Botha et al., 1998), peroxidases (van der Westhuizen et al., 1998b) and *β*-1,3-glucanases (van der Westhuizen et al., 1998a; van der Westhuizen et al., 2002), which accumulate in the apoplast of resistant plants within 24 hours of infestation (Botha et al., 1998). The exact function of these proteins in aphid defence remains unclear, but it has been suggested that chitinases might generate oligosaccharide elicitors from chitinous compounds released during aphid feeding (van der Westhuizen et al., 1998b). To assess whether the activation of HR-associated ROS differed between the NILs after *D. noxia* feeding, the activity of ROS enzymes was measured and DAB staining was performed to detect the presence of H_2_O_2_ in infested leaves. Staining with DAB revealed more oxidised deposits in the leaf tissue of the antibiotic Tugela-*Dn1* and to a lesser extent in the antixenotic Tugela-*Dn5* (Fig. 6). In Tugela-*Dn1*, thioredoxins are highly up-regulated when compared to the other NILs, these transcripts are known to be key in modulation of oxidative stress response (Vieira Dos Santos and Rey, 2006). However, little evidence of such a response is present in the susceptible Tugela and tolerant Tugela-*Dn2*. Higher levels of peroxidase activity were measured in the antibiotic Tugela-*Dn1* and antixenotic Tugela-*Dn5*, but not in the other NILs in response to feeding by *D. noxia* biotype SA1 (Fig. 2). Elevated levels of GST activity were also measured in the antibiotic Tugela-*Dn1* and antixenotic Tugela-*Dn5*, but not in the other NILs after infestation by *D. noxia* (Fig. 3). Interestingly, the enhanced LOX activity in the resistant plants as previously reported (Fig. 4) (Berner, 2006; Botha et al., 2010) was not observed in the present study. This may be ascribed to the fact that the host plants under study contained different resistant genes and were infested by different *D. noxia* biotypes. The timely generation of an oxidative burst and expression of ROS enzymes were prominent in Tugela-*Dn1* and Tugela-*Dn5* (van der Westhuizen et al., 1998a). Delayed (Tugela) or reduced (Tugela-*Dn2*, tolerant line) induction seems to be ineffective in initiating this kind of defensive strategy.

### The production of volatile organic compounds as the basis of antixenosis

Feeding by *D. noxia* characteristically leads to sealing off of transport elements, thereby allowing the aphid direct access to sap elements (Will and van Bel, 2006; Will et al., 2007). This process promotes apoplasmic and symplasmic isolation from the conducting elements, which may be the symptomatic causal effect of leaf rolling and streaking in susceptible varieties (Matsiliza and Botha, 2002; Saheed et al., 2007; Saheed et al., 2009). Even though the dubious resistance background of the antixenotic *Dn5* gene (Heyns et al., 2006) impairs accurate assessment of its effects during aphid feeding, differential expression of genes in the Tugela-*Dn5* NIL seem to link antixenosis with the fortification of the cell wall elements. Indeed, cell wall fortification and rapid deposition of callose in sieve pores has been viewed as an efficient wound response that seals off the pores in damaged phloem to prevent assimilate loss (Sjölund, 1997). The up-regulation of cellulase synthase (Richmond and Somerville, 2000) and CTP:phosphocholine cytidylyltransferase (Kent, 1997; Jackowski and Fagone, 2005) is thus not unexpected in the antixenotic Tugela-*Dn5*. However, antixenosis is mostly associated with the production of volatile organic compounds (VOCs). In the present study, the up-regulation of transcripts such as β-glucosidase (Mattiacci et al., 1995; Morant et al., 2008a; Morant et al., 2008b) and *O*-methyltransferase (Lam et al., 2007), associated with the production of VOCs were only differentially up-regulated in the antixenotic Tugela-*Dn5*, when compared with the other NILs. In *Pieris brassicae*, it was shown that β-glucosidases attractive to parasitic wasps (*Cotesia glomerata*) are produced in response to herbivory as defensive strategy (Mattiacci et al., 1995). *O*-methyltransferases on the other hand, constitute a large family of enzymes that methylate the oxygen atom of a variety of secondary metabolites including phenylpropanoids, flavonoids and alkaloids that result in VOC production (Lam et al., 2007). Further support for VOC production in a *Dn5* genetic background, comes from a study by Ni and Quisenberry (Ni and Quisenberry, 2000). In the latter, higher levels of cyclic hydroxamic acids (e.g., 2,4-dihydroxy-1,4-benzoxazin-3-one [DIBOA] and 2,4-dihydroxy-7-methoxy-1,4-benzoxazin-3-one [DIMBOA]) were found in the Betta-*Dn5* host plant, when compared with Betta NILs containing other *Dn* resistance genes (Ni and Quisenberry, 2000). Even though the authors could not conclusively tie the high levels of DIMBOA to the observed *D. noxia* resistance in the Betta-*Dn5* tested, DIMBOA nonetheless was shown in literature to contribute to plant resistance for both insect and pathogen pests in Gramineae (Niemeyer, 1988; Frey et al., 1997). DIMBOA has been reported to increase wheat resistance to pathogenic fungi (Weibull and Niemeyer, 1995) and five species of cereal aphids (Leszczynski et al., 1989; Leszczynski et al., 1995; Leszczynski and Dixon, 1990; Mayoral et al., 1994; Mayoral et al., 1996; Gianoli and Niemeyer, 1997).

### Tolerant plants exhibit up-regulated photosynthetic capacity

If the host is not able to activate an active defence syndrome, like in the case with the tolerant Tugela-*Dn2*, an alternative defensive strategy must be sourced. Chlorosis due to *D. noxia* infestation is thought to originate from interference with electron transport (Burd and Elliott, 1996; Haile et al., 1999; Heng-Moss et al., 2003; Botha et al., 2006). Susceptible wheat shows decreased levels of chlorophyll *a* upon infestation by *D. noxia* (Burd and Elliott, 1996; Ni et al., 2001; Wang et al., 2004b) which indicates damage to Photosystem I (PSI) (Botha et al., 2006). If this is indeed the case, it has serious implications for susceptible wheat under aphid attack. PSI catalyzes the electron transport from plastocyanin to ferredoxin (Haldrup et al., 2003). This reduced ferredoxin pool is mostly employed in generating NADPH for CO_2_ assimilation, but is also used in regulating the activity of, among others, CF_1_-ATP synthase and several enzymes in the Calvin cycle (Ruelland and Miginiac-Maslow, 1999). Under-reduced ferredoxin directly diminishes the plant's ability to synthesize ATP and carbohydrates. Increased photosynthetic capacity via up-regulation of photosystem components provides the most plausible mechanism for passive resistance against *D. noxia* feeding as observed in the present study. Evidence for ubiquitinylation (i.e., ubiquitin-specific protease) and up-regulation of transcripts *e.g.* chloroplast 50S ribosomal protein chlorophyll ab-binding protein, ATP-dependent Clp protease proteolytic subunit, malate synthase, fructose 1,6-bisphosphatase, and ferredoxin–thioredoxin reductase, all components of the photosynthetic machinery, in the tolerant Tugela-*Dn2* NIL, provides support for this passive resistance mechanism for aphid tolerance. Especially the fact that ferredoxin–thioredoxin reductases are only up-regulated in Tugela-*Dn2* provides supporting evidence for this form of resistance, since the latter enzymes are involved in the regulation of chloroplast photosynthetic enzymes (Arnér and Holmgren, 2000). Tolerant Betta-*Dn2* plants, for example, have very stable chlorophyll content during *D. noxia* feeding, suggesting that they can compensate for chlorophyll loss in some way (Heng-Moss et al., 2003).

**Fig. 7.**
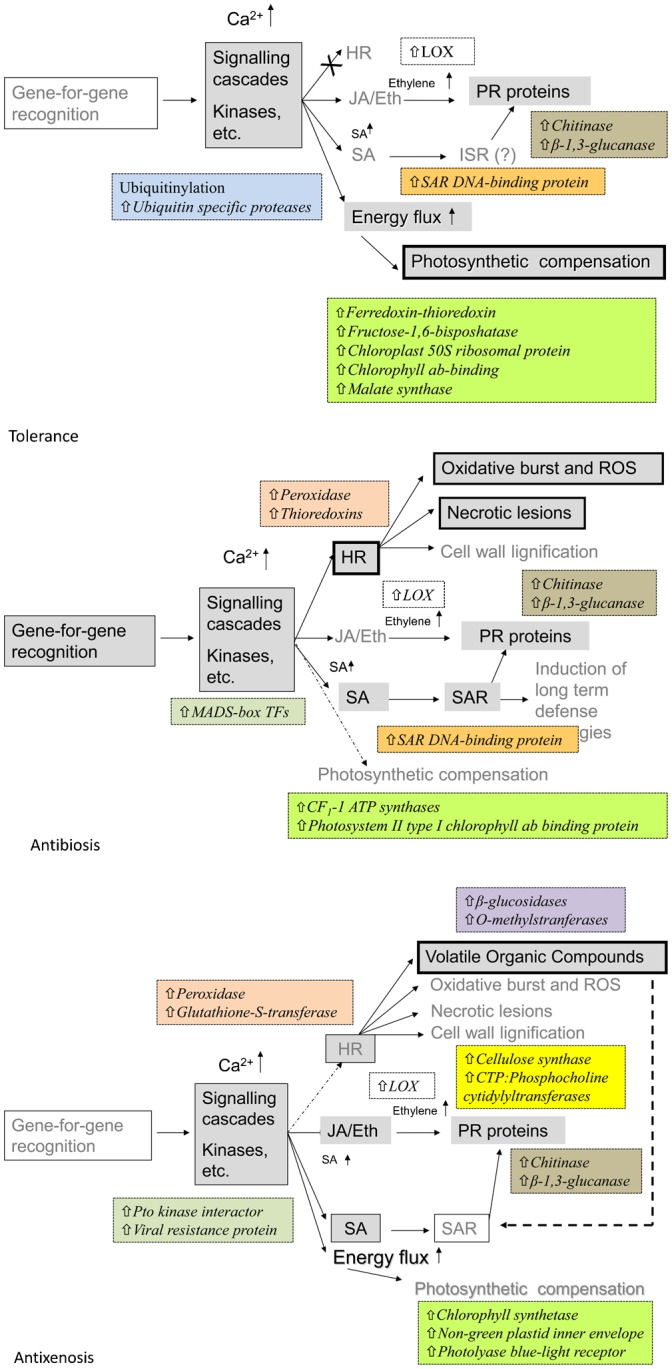
Models of the AAMPs in genotypes that express different modes of resistance according to Painter (Painter, 1951; Painter, 1958). Models are modified from Botha (Botha, 2013).

In conclusion, the patterns of differential gene expression of the three resistance NILs bred from the susceptible Tugela wheat line (Tolmay et al., 2006) are summarized in Fig. 7, and constitute a superb opportunity to study the effects of single resistance genes on an identical genetic background. From the evidence presented here, it is clear that initial aphid recognition in a *Dn* gene-specific manner coupled with the time and intensity of subsequent gene activation is critical in the eventual development of a resistant phenotype, whether an active antibiosis and antixenosis, or a passive photosynthetic compensatory tolerance.

## MATERIALS AND METHODS

### Plant material and aphid treatments

Hexaploid wheat (*Triticum aestivum* L.) germplasm of the near-isogenic lines (NILs) Tugela, Tugela-*Dn1* (Tugela*4/SA1684), Tugela-*Dn2* (Tugela*4/SA2199) and Tugela-*Dn5* (Tugela*4/SA463) was obtained from the Agricultural Research Council's Small Grain Institute (ARC-SGI), Bethlehem, South Africa (Liu et al., 2001; Tolmay et al., 2006). Seeds were sown into 5 pots for each cultivar and thinned to 3 seedlings per pot after 5 days. Plants were grown for 14 days (2–3 leaf stage) under greenhouse conditions in a 1:2:2:1 mixture of perlite (Chemserve, Olifantsfontein, South Africa), sifted bark compost, loam and sand at 25°C±2°C.

Aphids used for inducing plant responses were adult, apterous *Diuraphis noxia* of the South African biotype SA1, obtained from a colony established from field-collected parthenogenetic females at the ARC-SGI and maintained on the susceptible cv. Tugela.

For cDNA-AFLP analysis, plants of each cultivar were infested with 5 aphids and incubated for 2 h, 6 h, 12 h, or 24 h, while control plants remained uninfested. All leaves except the first leaf were harvested into liquid N_2_ and stored at −80°C prior to RNA isolation.

For Affymetrix analysis, plants of each cultivar were infested with 10 aphids and incubated for 48 h. All leaves except the first leaf were harvested into liquid N_2_ and stored at −80°C prior to RNA isolation.

For protein activity studies, plants were infested with 10 aphids as described above, but were incubated for 5 h, 24 h, 48 h and 144 h. Time intervals were chosen to capture events during the initial hypersensitive response (HR) and the long-term systemic acquired resistance (SAR) response.

### cDNA-AFLP analysis

Frozen leaf tissue was ground in liquid N_2_ using a mortar and pestle. Total RNA was extracted using a guanidine thiocyanate buffer method (Chomczynski and Sacchi, 1987) and the RNeasy kit with on-column DNase I digestion (Qiagen, Hilden, Germany). mRNA isolation was performed using the Qiagen Oligotex mRNA kit. cDNA synthesis was performed using the cDNA Synthesis System (Roche Applied Science, Mannheim, Germany) and the Qiagen MinElute Reaction Cleanup kit. Fifty nanograms of cDNA from each sample was used for cDNA-AFLP analysis (Bachem et al., 1996). cDNA-AFLP reactions were performed using the Expression Analysis kit (Li-Cor Biosciences, Lincoln NE, USA) according to the manufacturer's instructions for the generation of *Taq*I+0/*Mse*I+0 pre-amplification PCR products. These were assayed for yield and quality by 1% agarose gel electrophoresis. Using the approximate yield as a guide, the pre-amplification products were diluted accordingly in sterile dH_2_O and used as template for the final selective amplification. Selective amplifications were performed using sixteen *Mse*I+2 and *Taq*I+2 selective primer combinations from the kit. cDNA-AFLP profiles were separated on Li-Cor IR^2^ 4200S automated DNA sequencers using an 8% (v/v) LongRanger acrylamide gel solution (Cambrex Corp., East Rutherford NJ, USA) as previously described (Myburg et al., 2001). cDNA-AFLP images were saved in 16-bit TIFF format for image analysis.

Images generated on the Li-Cor DNA analyzers during electrophoresis were used to calculate band intensities of fragments judged to be differentially expressed, using the AFLP-QuantarPro software package (KeyGene Products B.V., Wageningen, Netherlands). Lane definitions, band scoring and sizing were carried out as described in the user's manual under default settings.

Statistical analysis of band intensity scores for differentially expressed transcript-derived fragments (TDFs) was carried out using Systat 7.01 (SPSS Inc., Chicago IL, USA) and Bioconductor in R (Gentleman et al., 2004). Statistical tests of differential expression were conducted using the moderated *t*-test in Bioconductor. Transcript-derived fragments (TDFs) with an absolute value of log_2_ fold change [log_2_(FC)]>1 and adjusted *P*-values of less than or equal to 0.05 between the different treatments, were considered differentially expressed. TDFs meeting this criterion were selected for further analysis by being excised from the gels, cloned and sequenced as previously described (Zaayman et al., 2009). Putative identities were assigned to TDFs by BLASTx and BLASTn similarity searches in GenBank (Altschul et al., 1997). Sequences were searched against the KEGG (Kanehisa and Goto, 2000) (http://www.genome.jp/kegg), BRENDA (Schomburg et al., 2002) (http://www.brenda-enzymes.info) and Gene Ontology (GO) databases (http://geneontology.org) using BLASTX via the program PLAN (He et al., 2007). Expectation values where E = 1e–02 and lower were considered significant. Cloned TDFs were assigned clone identification numbers of format AmoLve-*xx.xxx*, denoting an arbitrarily assigned two-digit primer code appended by the approximate band size in base pairs of the fragment as determined from cDNA-AFLP analysis.

### Confirmation of differential gene expression by RT-qPCR and RNA hybridization analyses

Real-time quantitative PCR (RT-qPCR) was performed on selected clones/probes occurring in both cDNA-AFLP and Affymetrix data sets to validate the expression obtained from transcript analysis. RT-qPCR was executed using the iScript One-Step RT-PCR Kit (Bio-Rad, Hercules CA, USA) and analysed using the iCycler iQ Real-Time PCR Detection Instrument (Bio-Rad). After primer design from the TDF sequence information using Primer Designer 5 (ver. 5.03, Scientific and Educational Software, Cary NC, USA), purified salt-free primers were synthesized (Integrated DNA Technologies, Coralville IA, USA). Five nanograms of total RNA and 10 µM of each primer (supplementary material Table S4) were used per reaction. All PCR reactions were carried out in triplicate. Relative quantification was done using the Tugela_0hpi (Tugela at 0 h post-infestation) sample as calibrator, and a serial dilution of the Tugela-*Dn1*_24hpi sample to generate the standard curve. The unregulated chloroplast 16S rRNA transcript was selected as endogenous control and used for normalization during relative quantification of target genes (Pfaffl, 2001).

Fifty nanograms of PCR product amplified using the primer sets employed in RT-qPCR served as template for the synthesis of fluorescein-11-dUTP-labeled probes using the Gene Images Random Prime Labeling kit (GE Life Sciences, Uppsala, Sweden) according to the manufacturer's instructions. Incorporation of the fluorescein label was monitored by comparing the fluorescence with a reference strip of serially diluted nucleotide mix containing the fluorescein-11-dUTP molecules. Two hundred nanograms of RNA from each sample was blotted onto Hybond-N+ nylon membrane (GE Life Sciences) using the BioDot-SF device (Bio-Rad) according to the recommendations in the user manual. The RNA was cross-linked to the membrane using the UVIlink CL508 ultraviolet crosslinker (UVItec Ltd., UK) set at 0.240 J for 3 min. Probe hybridization was performed in a Techne HB-1D hybridization chamber (Techne Inc., Burlington NJ, USA). Pre-hybridization of RNA was performed at 65°C in 0.125 ml•cm^−2^ hybridization buffer for 30 min, whereafter the denatured probe was added and allowed to hybridize overnight at 65°C. Probe detection was carried out with the Gene Images CDP-Star Detection kit (GE Life Sciences) according to the instructions of the manufacturer. Blots were visualized by exposure to Amersham Hyperfilm ECL chemiluminescence film (GE Life Sciences) overnight.

### GeneChip Wheat Genome Array (Affymetrix, USA) analysis

Frozen leaf tissue was ground in liquid N_2_ using a mortar and pestle, and then incubated in PureLink Plant RNA purification Reagent (Invitrogen, USA) at room temperature for 10 minutes. Total RNA was extracted using the Qiagen RNeasy Plant Mini Kit with on-column DNase I digestion following the manufacturer's instructions. Integrity and quantity of the RNA was tested using Experion RNA StdSen Chips (Bio-Rad). The RNA samples were sent to the Centre for Proteomic and Genomic Research (CPGR, Cape Town, South Africa), where additional quality control and subsequent RNA labeling, processing, and data gathering were performed, according to Affymetrix protocols. A total of 12 samples were hybridized to arrays. The experimental design enabled for a complete comparison between all treatments at the specific time point and enabled direct pairwise comparisons between all the treatments. Different quality control checks were performed including inspection of hybridized images, boxplots and histograms of log_2_(PM) values, examination of hybridization and Poly(A) controls. Data analysis was carried out using Bioconductor in R (Gentleman et al., 2004). Data preprocessing and summarization were performed using Robust Multichip Average (RMA) (Irizarry et al., 2003), Affymetrix Microarray Suite 5 (MAS5.0) (Harr and Schlötterer, 2006), GeneChip Robust Multichip Average (GCRMA) (Zakharkin et al., 2005), Variance Stabilisation (VSN) (Huber et al., 2002) and Probe Level Models (PLM) (Bolstad et al., 2003). Only expression data significant to all normalization methods were included in further analyses. Statistical tests of differential expression were conducted using the moderated *t*-test through the limma (Linear Models for Microarrays) package in Bioconductor by comparing differential expression between treatments obtained after normalizations as follows: Tugela infested ↔ Tugela-*Dn1* infested ↔ Tugela-*Dn2* infested ↔ Tugela-*Dn5* infested. The Benjamini-Hochberg multiple testing adjustment was applied in order to control the comparison-wise false discovery rate (Benjamini and Hochberg, 1995). After analyses of the different data sets, the obtained data sets were saved in Excel spreadsheet format. Perl scripts (http://www.perl.org) were written and run in the Unix environment to enable direct comparison between the different data sets for the identification of genes that were differentially expressed after normalization (Swanevelder, 2010).

Genes corresponding to probe sets with an absolute value of log_2_ fold change [log_2_(FC)]>1 and adjusted *p*-values of less than or equal to 0.05 were considered differentially expressed. The target sequences corresponding to genes identified as differentially expressed were obtained from Affymetrix. Target sequences were then searched against the KEGG (Kanehisa and Goto, 2000) (http://www.genome.jp/kegg), BRENDA (Schomburg et al., 2002) (http://www.brenda-enzymes.info) and Gene Ontology (GO) databases (http://geneontology.org) using BLASTX via the program PLAN (He et al., 2007). Additionally, BLAST2GO (http://www.blast2go.com/start-blast2go) was used to obtain the putative Gene Ontology (GO) (Conesa and Götz, 2008). Annotation was obtained for the top significant hit (using an E-value cutoff of 1e–10) for each target sequence. Venn diagrams were drawn using Venn diagram software tools (http://bioinformatics.psb.ugent.be/webtools/Venn).

### Protein activity assays

Extraction of enzymes was performed using a modified method of Rao et al. (Rao et al., 1997). Leaf tissue was snap frozen in liquid N_2_ and then ground to powder, whereafter 500 µl of ice cold 100 mM potassium phosphate buffer (pH 7.5) containing 1 mM ethylenediaminetetraacetic acid (EDTA) and 1% polyvinylpyrrolidone (PVP) was added. After centrifugation (25,000 g, 20 min, 4°C), the supernatant was used for enzyme assays. All enzyme activities were conducted in triplicate using independent biological repeats.

Peroxidase activity was determined following a modified method of Zieslin and Ben-Zaken (Zieslin and Ben-Zaken, 1991). The assay solution contained 0.1 M sodium phosphate buffer (pH 5), 3 mM H_2_O_2_, 3 mM guaiacol and an aliquot of the enzyme extract. The formation of tetraguaiacol was monitored at 470 nm. Peroxidase activity was expressed as mmol tetraguaiacol•min^−1^•mg^−1^ protein.

Gluthathione-*S*-transferase (GST) enzyme activity was measured as described (Venisse et al., 2001). The assay solution contained 0.1 M phosphate buffer (pH 6.5), 3.6 mM reduced glutathione, 1 mM 1-chloro-2,4-dinitrobenzene and an aliquot of the enzyme extract. The formation of GS-DNB conjugate was monitored at 340 nm. GST activity was expressed as mmol GSH•min^−1^•mg^−1^ protein.

Lipoxygenase (LOX) activity was measured according to the methods of Grossman and Zakut (Grossman and Zakut, 1979) and Ocampo et al. (Ocampo et al., 1986). The LOX reaction mixture contained 0.1 M sodium citrate-phosphate buffer (pH 6.2), 2.5 mM linoleic acid and an aliquot of the enzyme extract. The formation of hydroperoxyoctadecadienoic acid (HPOD) was monitored at 234 nm. LOX activity was expressed as nmol HPOD•min^−1^•mg^−1^ protein.

The colorimetric assay of *β*-1,3-glucanase was carried out according to a modified method of Fink et al. (Fink et al., 1988). An aliquot of the enzyme was incubated with 0.5 ml substrate, laminarin (1 mg•ml^−1^ 50 mM sodium acetate buffer, pH 4.5) at 37°C for 10 min. Subsequently, 0.5 ml reagent of Somogyi (Somogyi, 1952) was added and the mixture heated at 100°C for 10 min. After cooling and the addition of 0.5 ml of arsenomolybdate reagent of Nelson (Nelson, 1944), absorbance of the coloured product was measured at 540 nm. A standard curve relating the amount of glucose equivalents to absorbance (A_540_) was employed for the determination of enzyme activity. The formation of glucose was a linear function of enzyme concentration extracted (data not shown). *β*-1,3-glucanase activity was expressed as mg glucose•min^−1^•mg^−1^ protein.

Protein concentration was determined according to the method of Bradford (Bradford, 1976) using the Bio-Rad protein assay reagent with bovine albumin (Bio-Rad) as a standard. The Glomax Spectrophotometer, following the method described by Rylatt and Parish, was used for this purpose (Rylatt and Parish, 1982).

Enzyme activity measurements were analyzed by analysis of variance (ANOVA) by using a split-plot model with genotype as the main plot. This model was used to achieve greater precision in comparing treatments and finding differential treatment effects relative to the genotype (interaction). Comparisons between two treatment means in the same genotype were made using the least significant difference test (*α* = 0.05). The Student-Newman-Keuls test (Newman, 1939) was applied to conduct multiple comparisons of the treatments. Statistical analyses were conducted using SAS (SAS Institute, 1988).

### DAB staining for the presence of H_2_O_2_

DAB staining was performed according to the protocol of Thordal-Christensen et al. (Thordal-Christensen et al., 1997). The fourth leaves from four independent biological replicates per treatment were collected 6 days after aphid infestation. Feeding aphids were removed from all leaves with a paintbrush, and leaves were placed in 1 mg•ml^−1^ 3,3′-diaminobenzidine (DAB)-HCl, pH 3.8, (Sigma–Aldrich, St. Louis, MO, USA) and incubated overnight in the dark at room temperature with gentle agitation. The tissues were subsequently cleared in 75% ethanol at 37°C with gentle agitation for 5 h, replacing the ethanol as needed. The presence of H_2_O_2_ is revealed by reddish-brown polymerized deposits.

## Supplementary Material

Supplementary Material
