# Supplementary material for: Near-isogenic lines of Triticum aestivum with distinct modes of resistance exhibit dissimilar transcriptional regulation during Diuraphis noxia feeding
Source: Biol Open. 2014 Oct 31;3(11):1116–26. doi: 10.1242/bio.201410280 (PMC4232770; doi:10.1242/bio.201410280)
Supplement: Supplementary Material [file supp_3_11_1116__index.html]

Near-isogenic lines of Triticum aestivum with distinct modes of resistance exhibit dissimilar transcriptional regulation during Diuraphis noxia feeding — Supplementary Material 

# Near-isogenic lines of *Triticum aestivum* with distinct modes of resistance exhibit dissimilar transcriptional regulation during *Diuraphis noxia* feeding

## bio.201410280 Supplementary Material

**Files in this Data Supplement:**

- Supplementary Material - Anna-Maria Botha et al. doi: 10.1242/bio.201410280
- Table S2 - **Genes significantly regulated after *D. noxia* infestation.** Indicated are sequence name, description and length, number of hits, mean e-value and Gene Ontology (http://www.blast2go.com/start-blast2go).
- Table S3 - **Genes significantly up- and down-regulated between NiLS after normalization with MAS5, RMA, GCRMA, PLM and VSN.** Indicated is the GenBank accession number, Affymetrix probe set ID and target description. Also indicated is LogFC, average expression, *p*-value, adjusted *p*-value (Benjamini and Hochberg, 1995), and gene expression. Red = up-regulation; green = down-regulation.
