# Supplementary material for: Near-isogenic lines of Triticum aestivum with distinct modes of resistance exhibit dissimilar transcriptional regulation during Diuraphis noxia feeding
Source: Biol Open. 2014 Oct 31;3(11):1116–26. doi: 10.1242/bio.201410280 (PMC4232770; doi:10.1242/bio.201410280)
Supplement: Supplementary Material [file supp_bio.201410280_bio.201410280-s1.pdf]

Biology Open (2014) 000, 1–11 doi:10.1242/bio.201410280

Supplementary Material

Anna-Maria Botha et al. doi: 10.1242/bio.201410280

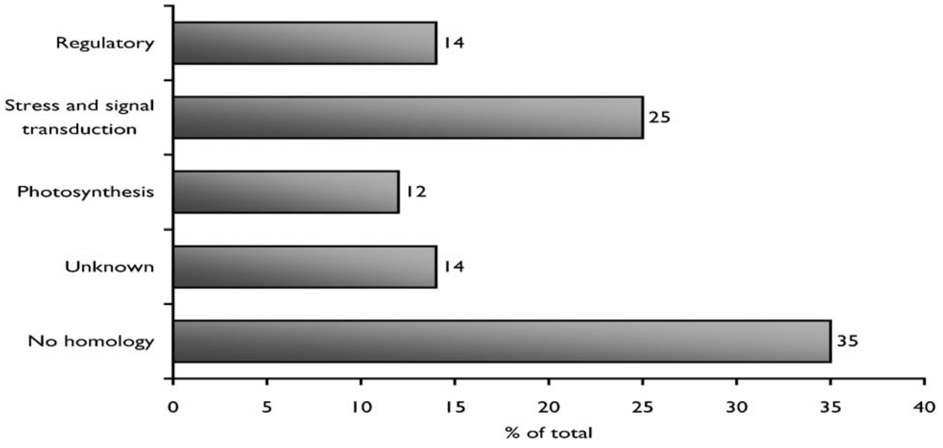

Fig. S1. Percentage contribution of each functional category of assigned TDFs to the total number of obtained TDFs after classification into broad functional categories (i.e., regulatory; stress and signal transduction; photosynthesis; unknown and no homology). Numbers of obtained TDFs are indicated on the bars. Classification of putative TDF identities are based on BLASTx and BLASTn results after excision and cloning of cDNA-AFLP fragments (Altschul et al., 1997). TDFs classed as unknown demonstrate significant homology to sequences for proteins with unknown function. TDFs with no homology had no significant similarity to any database sequences via BLASTx or BLASTn.

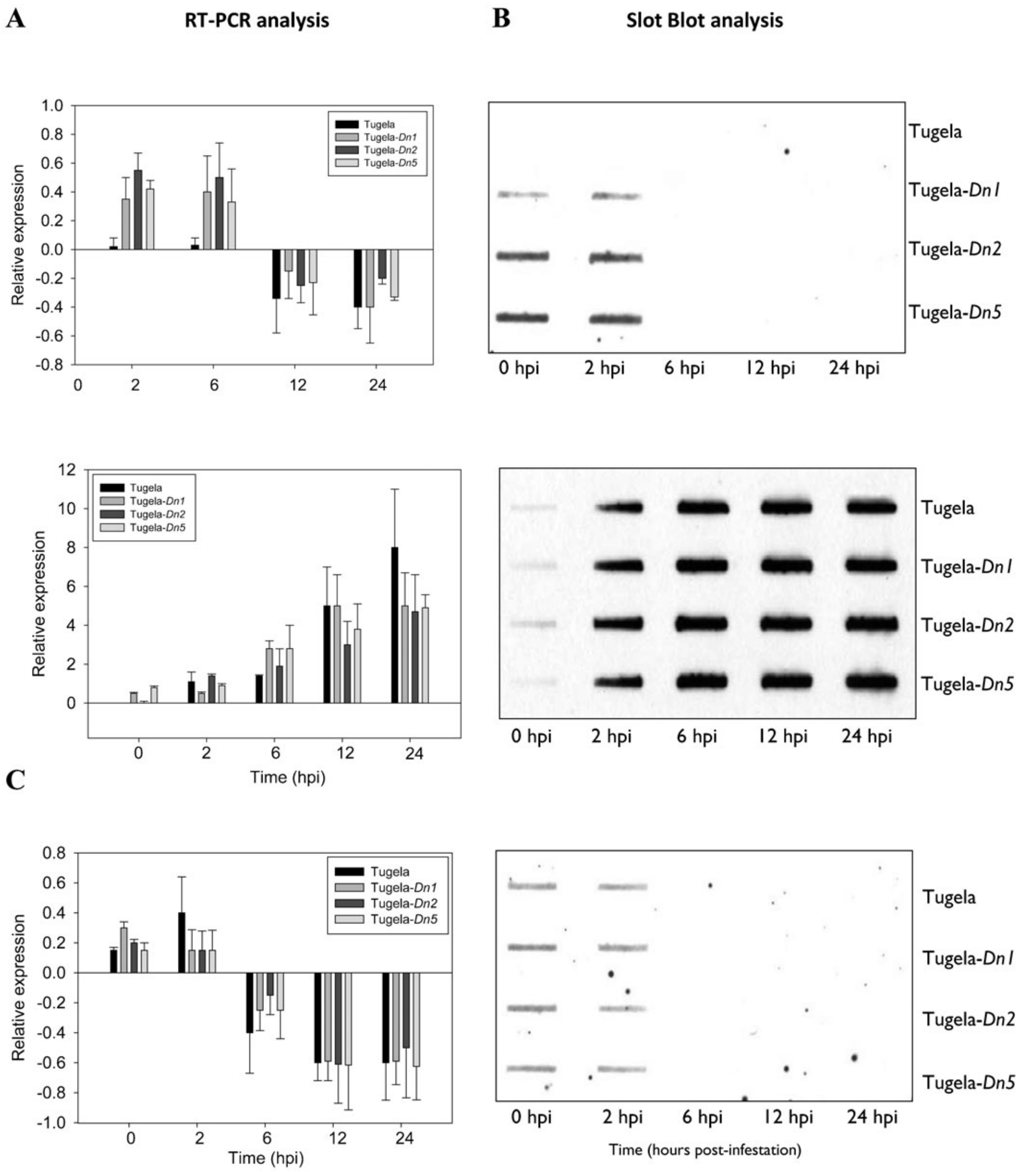

Fig. S2. Comparison of expression profiles of wheat transcripts differentially regulated during aphid infestation obtained using qRT-PCR and RNA hybridization, where the expression level is calculated relative to the expression level of the Tugela_0hpi sample and is normalized to the expression of the unregulated chloroplast 16S rRNA transcript. (A) Stress related-like protein interactor; (B) KCOI potassium channel; (C) Inorganic pyrophosphatase.

Biology Open (2014) 000, 1–11 doi:10.1242/bio.201410280

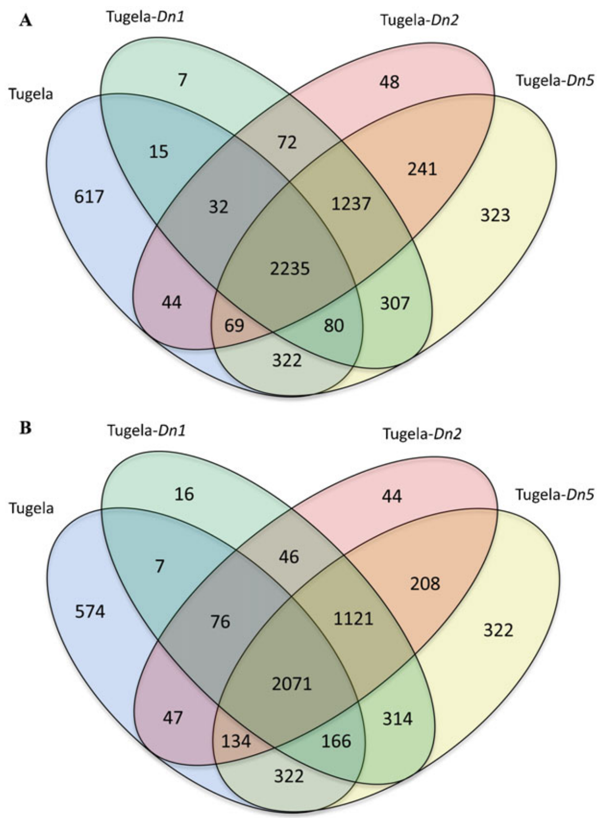

Fig. S3. Venn diagram of genes significantly down- (A) and up-regulated (B) in NiLs after *D. noxia* infestation. Genes represented were significantly up- and down-regulated after normalization ($\log_2$ fold change, $P<0.001$).

Biology Open

Biology Open (2014) 000, 1–11 doi:10.1242/bio.201410280

Table S1. Sequenced derived transcripts to which putative identity could be assigned following BLASTn and BLASTx searches (Altschul et al., 1997)

Functional group and putative identity				
Accession	Clone ID	Size (bp)	Homology	E value
**Regulatory**				
BAD36693	AmoLve-26.340	657	Lingual lipase-like [Oryza sativa[a]]	$5e^{-41}$
CAD66657	AmoLve-45.211	215	TPA putativecysteine protease [Hordeum vulgare subsp. vulgare[a]]	$3e^{-24}$
BAD33181	AmoLve-56.112	115	Putative transfactor [Oryza sativa[a]]	$1e^{-02}$
AAY90056	AmoLve-66.371	377	Ubiquitin [Triticum aestivum[a]]	$2e^{-23}$
ABA97973.2	AmoLve-68.344	390	Putative E3 ubiquitin-proteinligase 1 [Oryza sativa[a]]	$6e^{-39}$
AAD46404	AmoLve-88.234	243	Ethylene-responsive RNA helicase [Lycopersicon esculentum[a]]	$4e^{-15}$
BAB34228	AmoLve-88.250	252	Hypothetical C4-type zinc finger protein TraR-family [Escherichia coli O157:H7[a]]	$1e^{-34}$
**Stress and signal transduction**				
BAD44784	AmoLve-11.250	257	Putative mitochondrial half-ABC transporter [Oryza sativa[a]]	$1e^{-14}$
AAS07290	AmoLve-11.270	323	Putative mechano-sensitive ion channel protein [Oryza sativa[a]]	$5e^{-14}$
BAD10571	AmoLve-11.450	454	GHMP kinase-like protein [Oryza sativa[a]]	$2e^{-57}$
AAX19515	AmoLve-22.210	248	Serine/threonine protein kinase domain [Triticum aestivum[a]]	$9e^{-14}$
BAD08086	AmoLve-22.225	223	Inorganic pyrophosphatase [Oryza sativa[a]]	$2e^{-30}$
BAD17253	AmoLve-26.245	249	Putative UDP-glucose glucosyltransferase 1 [Oryza sativa[a]]	$6e^{-15}$
ABA91061	AmoLve-26.250.1	257	Clathrin heavy chain [Oryza sativa[a]]	$4e^{-28}$
BAD09693	AmoLve-26.265	263	Putative stress related-like protein interactor [Oryza sativa[a]]	$7e^{-35}$
AF262979	AmoLve-45.323	322	PDI-1 proteindisulfideisomerase 1 [Triticum aestivum[b]]	$3e^{-149}$
AY770627	AmoLve-61.272	338	Outward-rectifying potassium channel (KCO1) mRNA [Hordeum vulgare subsp. vulgare[b]]	$4e^{-16}$
BAB55540	AmoLve-66.172	300	Putative 66 kDa stress protein [Oryza sativa[a]]	$1e^{-40}$
BAD05751	AmoLve-88.300	166	IDI2 isopentenyl-diphosphate delta isomerase 2 [Hordeum vulgare subsp. vulgare[a] BAB21393]	$3e^{-46}$
**Photosynthesis**				
NP_566086	AmoLve-11.445	448	TMP 14 kDa thylakoid membrane phosphoprotein [Arabidopsis thaliana[a]]	$2e^{-14}$
CAG25595	AmoLve-66.306	199	Putative Rubisco small subunit [Triticum turgidum subsp. durum[a]]	$4e^{-07}$
CAG25595	AmoLve-66.319	155	Putative Rubisco small subunit [Triticum turgidum subsp. durum[a]]	$4e^{-07}$
BAD05751	AmoLve-66.323	394	Putativeaconitatehydratase [Oryza sativa[a]]	$3e^{-46}$
CAG25595	AmoLve-86.322	324	Putative Rubisco small subunit [Triticum turgidum subsp. durum[a]]	$4e^{-07}$
CAB46084	AmoLve-88.303	305	Fructose-1,6-bisphosphatase [Pisum sativum[a]]	$8e^{-44}$
**Unknown**				
AAS07252	AmoLve-26.250	255	Expressed protein [Oryza sativa[a]]	$5e^{-33}$
XM_473940	AmoLve-43.129	265	OSJNBb0085C12.17 [Oryza sativa[a]]	$4e^{-14}$
AL731597	AmoLve-45.370	375	OSJNBa0023J03.10 [Oryza sativa[a]]	$4e^{-30}$
BAF11326.1	AmoLve-61.374	388	Os03g0221300 [Oryza sativa[a]]	$3e^{-09}$
EAZ25741	AmoLve-66.244.1	340	OsJ_009224 [Oryza sativa[a]]	$1e^{-03}$
AL606658.5	AmoLve-68.292	334	OSJNBb0016D16.15 [Oryza sativa[a]]	$3e^{-05}$
EAZ14041	AmoLve-88.231	144	OsJ_003866 [Oryza sativa[a]]	$8e^{-17}$
**No homology**				
EL563871	AmoLve-11.340	345	No match[a,b]	
EL563872	AmoLve-22.270	281	No match[a,b]	
EL563873	AmoLve-22.275	322	No match[a,b]	
EL563874	AmoLve-26.220	215	No match[a,b]	
EL563875	AmoLve-26.270	257	No match[a,b]	
EL563876	AmoLve-26.355	336	No match[a,b]	
EL563877	AmoLve-42.151	194	No match[a,b]	
EL563878	AmoLve-42.321	324	No match[a,b]	
EL563879	AmoLve-43.139	180	No match[a,b]	
EL563880	AmoLve-45.229	235	No match[a,b]	
EL563881	AmoLve-66.169	170	No match[a,b]	
EL563882	AmoLve-67.181	217	No match[a,b]	
EL563883	AmoLve-67.216	218	No match[a,b]	
EL563884	AmoLve-68.268	292	No match[a,b]	
EL563885	AmoLve-68.407	410	No match[a,b]	
EL563886	AmoLve-86.113	114	No match[a,b]	
EL563887	AmoLve-88.189	219	No match[a,b]	

Functional categories, GenBank accession numbers, clone identities, fragment sizes and E values are indicated.
[a]Similarity analysis of TDF sequence using BLASTx.
[b]Similarity analysis of TDF sequence using BLASTn.

Table S2. Genes significantly regulated after *D. noxia* infestation. Indicated are sequence name, description and length, number of hits, mean e-value and Gene Ontology (http://www.blast2go.com/start-blast2go). See supplementary webpage.

Table S3. Genes significantly up- and down-regulated between NiLS after normalization with MAS5, RMA, GCRMA, PLM and VSN. Indicated is the GenBank accession number, Affymetrix probe set ID and target description. Also indicated is LogFC, average expression, *p*-value, adjusted *p*-value (Benjamini and Hochberg, 1995), and gene expression. Red = up-regulation; green = down-regulation. See supplementary webpage.

Table S4. Primer combinations used in qRT-PCR

Clone ID	Identity	Primer A	Primer B
AmoLve-22.225	Inorganic pyrophosphatase	ACCGTCACTTCAGAGACATC	GCTGGGAGGAAATCATTCAC
AmoLve-26.265	Stress related-like protein interactor	CCTTGGTTGGTGACACATTC	GGTTCAGCCCATTTCTTTGC
AmoLve-61.272	KCOI potassium channel	GTATTCGCCTGATGGAAC	CTGCCTTCACTGGATAAC
